# Safety profile and pharmacokinetic analyses of the anti-CTLA4 antibody tremelimumab administered as a one hour infusion

**DOI:** 10.1186/1479-5876-10-236

**Published:** 2012-11-21

**Authors:** Antoni Ribas, Jason A Chesney, Michael S Gordon, Amy P Abernethy, Theodore F Logan, David H Lawson, Bartosz Chmielowksi, John A Glaspy, Karl Lewis, Bo Huang, Erjian Wang, Poe-Hirr Hsyu, Jesus Gomez-Navarro, Diana Gerhardt, Margaret A Marshall, Rene Gonzalez

**Affiliations:** 1 Division of Hematology-Oncology, 11-934 Factor Building, Jonsson Comprehensive Cancer Center at the University of California Los Angeles, Los Angeles, CA 90095-1782, USA; 2 James Graham Brown Cancer Center, University of Louisville, Louisville, KY, USA; 3 Premiere Oncology of Arizona, Scottsdale, AZ, USA; 4 Duke University, Durham, NC, USA; 5 Indiana University, Indianapolis, IN, USA; 6 Emory University, Atlanta, GA, USA; 7 University of Colorado, Aurora, CO, USA; 8 Pfizer Inc., Groton, CT, La jolla, CA, USA; 9 Millennium-Takeda, Boston, Massachusetts, USA

**Keywords:** Melanoma, Tremelimumab, CTLA4

## Abstract

**Background:**

CTLA4 blocking monoclonal antibodies provide a low frequency but durable tumor responses in patients with metastatic melanoma, which led to the regulatory approval of ipilimumab based on two randomized clinical trials with overall survival advantage. The similarly fully human anti-CTLA4 antibody tremelimumab had been developed in the clinic at a fixed rate infusion, resulting in very prolonged infusion times. A new formulation of tremelimumab allowed testing a shorter infusion time.

**Methods:**

A phase 1 multi-center study to establish the safety and tolerability of administering tremelimumab as a 1-hour infusion to patients with metastatic melanoma. Secondary endpoints included pharmacokinetic and clinical effects of tremelimumab.

**Results:**

No grade 3 or greater infusion-related adverse events or other adverse events preventing the administration of the full tremelimumab dose were noted in 44 treated patients. The overall side effect profile was consistent with prior experiences with anti-CTLA4 antibodies. Objective tumor responses were noted in 11% of evaluable patients with metastatic melanoma, which is also consistent with the prior experience with CTLA4 antagonistic antibodies.

**Conclusions:**

This study did not identify any safety concerns when tremelimumab was administered as a 1-hour infusion. These data support further clinical testing of the 1-hour infusion of tremelimumab. (Clinical trial registration number NCT00585000).

## Background

The cytotoxic T lymphocyte-associated antigen 4 (CTLA4) is a co-inhibitory receptor transiently expressed on the surface of activated T lymphocytes and constitutively expressed by T regulatory (Treg) cells [1]. It has a dominant immune-dampening effect on self-reactive T cells. CTLA4 has become a validated target for therapy in patients with metastatic melanoma based on the survival advantage in two phase 3 clinical trials using the CTLA4 blocking fully human IgG1 antibody ipilimumab (formerly MDX010) [2,3]. Tremelimumab (also known as CP-675,206) is a fully human monoclonal antibody of IgG2 subtype that also binds and antagonizes the function of CTLA4. The antagonistic effect of tremelimumab on CTLA4 enhances human T cell activation as demonstrated using *in vitro* assays [4]. This antibody has been in clinical testing for the treatment of malignancies since 2002 [5], and continues in clinical development as single agent and in combination in several cancer indications.

Prior clinical trials demonstrated that tremelimumab induces durable tumor regressions, sometimes lasting beyond 5 years, in approximately 10% of patients with metastatic melanoma [5-7]. These tumor responses are mediated by the intratumoral infiltration of cytotoxic T lymphocytes (CTLs) as demonstrated in patient-derived tumor biopsies [8,9]. The most common toxicities with tremelimumab administration are skin rash and diarrhea/colitis, with a few percent of patients experiencing endocrine abnormalities such as thyroiditis and hypophysitis. Grade 3/4 toxicities occur in about 15-20% of patients. A randomized phase 2 clinical trial compared the regimens of 10 mg/kg monthly and 15 mg/kg every 90 days. This study suggested that the 15 mg/kg every three month schedule was preferred based on lower toxicities while maintaining tumor response rates and having similar survival [6]. This dosing regimen has been tested in two pivotal clinical trials in patients with advanced melanoma. A phase 2 single arm trial in patients (n=251) with previously treated metastatic melanoma demonstrated a tumor response rate of 9.1% per investigator assessment and 6.6% per central radiological review, and thus failed to demonstrate that the response rate exceeds 10% [10]. In a phase 3 randomized clinical trial, the median overall survival was 12.6 months in the tremelimumab arm compared to 10.7 months in the chemotherapy arm, but the difference in overall survival was not statistically significant. A major contributing factor was a higher than expected use of ipilimumab in patients randomized to the chemotherapy control arm. Response rates in the two arms were similar, but duration of response was significantly longer in the tremelimumab arm [7], consistent with the notion that this agent provides durable tumor responses in a subset of patients as its major clinical benefit.

Prior to this study, tremelimumab had been administered to over 800 subjects at a fixed rate of 100 mL/hour, resulting in infusion times between 1.0 and 5 hours. Since tremelimumab is a fully human monoclonal antibody that did not demonstrate evidence of infusion-related cytokine release in prior clinical testing [5,6,10],, nor there were reports of infusion reactions with ipilimumab [2,3,11], it was reasoned that a shorter infusion time may be safe. A new formulation of tremelimumab allowed a lower fluid volume for administration, facilitating a shorter infusion time. The current study was conducted to establish the safety and tolerability of administering tremelimumab as a 1-hour infusion, which may increase patient convenience in further clinical testing of this antibody.

## Materials and methods

### Study design and conduct

This open-label, single arm multi-institutional study was planned for approximately 110 subjects. The original study protocol stated that enrollment should terminate if 3 or more subjects experienced tremelimumab-related adverse events (AEs) during or immediately after the infusion. All subjects signed a written informed consent approved by the Institutional Review Board (IRB) at each study site. The study was conducted in accordance with local regulations, the guidelines for Good Clinical Practice (GCP), and the principles of the current version of the Declaration of Helsinki. The study opened to accrual at 7 US centers, was sponsored by Pfizer Inc. (New York, NY), and had the clinical trial registration number NCT00585000.

### Study objectives and assessments

The primary objective was to assess the safety and tolerability of tremelimumab at 15 mg/kg as a one-hour infusion. Secondary objectives included characterizing the pharmacokinetics (PK), monitoring for human anti-human antibody (HAHA), and assessing anti-tumor activity by best overall tumor response rate using Response Evaluation Criteria in Solid Tumors (RECIST) criteria.

### Study population

Eligible subjects were aged 18 years or older with histologically confirmed stage III or IV melanoma considered to be surgically incurable, with evidence of at least 1 measurable or non-measurable lesion according to RECIST criteria, with an Eastern Cooperative Oncology Group (ECOG) performance status of 1 or lower, with adequate bone marrow, hepatic, and renal function. Subjects were excluded if they had had a potential requirement for systemic corticosteroids or concurrent immunosuppressive drugs, had an inherited or acquired immunodeficiency, had a history of chronic autoimmune disease, inflammatory bowel disease, active or chronic viral hepatitis, uveitis or melanoma-associated retinopathy. Patients with a history of brain metastases were eligible if they had been adequately treated with surgery or stereotactic radiosurgery and were stable for at least 3 months prior to enrollment.

### Tremelimumab administrations

Subjects received an intravenous administration of tremelimumab at a dose of 15 mg/kg via infusion pump as a 1-hour infusion, repeated every 90 days up to a maximum of 4 doses.

### Safety, efficacy, and pharmacokinetic evaluations

Adverse events (AEs) were graded according to the National Cancer Institute Common Terminology Criteria for Adverse Events (CTCAE Version 3.0). Radiologic and clinical evaluations were performed within 28 days prior to dosing and within 10 days prior to the planned start of each 90-day cycle. Blood samples for pharmacokinetic analyses were obtained just prior to administration of tremelimumab for every treatment cycle (cycles 1–4), with multiple additional samples taken in cycle 1 (1 and 6 hours after the end of infusion, on days 2, 3, 8, 15, 30, 45, 60 and 75 with ± 2 days to accommodate scheduling problems) to fully characterize the pharmacokinetic profile of the infusion regimen.

### Pharmacokinetic analysis

Plasma samples were analyzed for tremelimumab concentrations using a validated, sensitive and specific enzyme-linked immunosorbent assay (ELISA) (ALTA Analytical Laboratory, San Diego, CA) as previously described [5,6]. The PK assay underwent formal methodology optimization, with linear calibration of standard responses over the range of 7.8 to 250 ng/mL using a 5 parameter curve-fit weighted model. Those samples with concentrations above the upper limits of quantification were adequately diluted into the calibration range. The lower limit of quantification (LLOQ) for tremelimumab was 7.8 ng/mL. Assay precision, expressed as the between-day coefficients of variation (CV [%]) of the mean estimated concentrations of quality control samples ranged from 9.34% to 11.5% for low (25.0 ng/mL), medium (50.0 ng/mL), high (100 ng/mL), and diluted (25.0 ng/mL) concentrations. Samples below the LLOQ were set to 0 ng/mL for analysis. Nominal sample collection times were used for the PK analysis. The concentration-time data were analyzed by non-compartmental analysis. Parameters of tremelimumab disposition included clearance (CL), volume of distribution at steady state (V_ss_), and terminal phase half-life (t_1/2_). Parameters of tremelimumab exposure included plasma concentration of tremelimumab one hour after the end of infusion (C_max_) and area under the concentration curve from time zero to infinity (AUC_inf_). Comparison PK data is presented from study A3671008, a pivotal phase II study in which tremelimumab was administered as a 5-hour infusion (11).

### Human anti-human antibody (HAHA) assessment

A blood specimen for HAHA was obtained just prior to administration of tremelimumab every treatment cycle. In cycle 1, blood specimens were also obtained on days 30 and 60. HAHA samples were analyzed for the presence or absence of anti-tremelimumab antibodies following a tiered approach using screening, confirmation and titer/quantitation as previously described [5]. The semi-quantitative ELISA with electrochemiluminescence detection (ECL) included a positive control (cynomolgus anti-CP675,206 antibody sera) and negative control (pooled normal human plasma). Assay precision, expressed as the between-day CV%, was less than 16.7% for the positive control (used to calculate assay cut point) and 13.1% for the negative control.

### Statistical analysis

The primary goal of the study was to demonstrate that the infusion-related reaction rate for one-hour infusion did not exceed 5% with a one-sided exact binomial test at 10% significance level (alpha=0.10). 110 subjects would provide 90% power to reject the null hypothesis (infusion-related reaction rate ≥5%) when the true infusion-related reaction rate did not exceed 1%.

## Results and discussion

### Study subjects and treatment administration

Forty-nine subjects were enrolled to this protocol between December 2007 and July 2008, and 44 subjects received at least one infusion of tremelimumab. Enrollment to this study was stopped early by the sponsor. Forty-one subjects were included in the analysis of laboratory data; 3 subjects were excluded as they had no laboratory data recorded after day 0 or day 1 (two subjects progressed rapidly and one withdrew consent). Demographic characteristics are summarized in Table 1. Seventy percent were male and 70% were older than 65 years. The mean duration since first diagnosis of malignant melanoma was 5.3 years (range 0.1 to 20.4 years). Over half of the patients had stage IV M1c disease and approximately 80% had previously received at least 1 prior systemic therapy for advanced melanoma. Twenty-nine (65.9%) treated patients received a single dose; 6 (13.6%) received a total of 2 doses; 3 (6.8%) received a total of 3 doses, and six (14%) subjects completed 4 cycles of treatment. Four (9.1%) subjects had at least one dose delay and three (7%) had the therapy discontinued due to an adverse event beyond the time of infusion (one each due to autoimmune thrombocytopenia, allergic encephalitis and grade 3 diarrhea).

**Table 1:** Demographic characteristics

**Number (%) of Subjects**	**(N=44)**
Sex	
Male	31 (70.5)
Female	13 (29.5)
Age (years)	
Mean (SD)	57.8 (12.3)
Range	26-80
Age Category	
<65	31 (70.5)
≥65	13 (29.5)
Race	
White	41 (93.2)
Black	2 (4.5)
Asian	1 (2.3)
ECOG performance status	
0	30 (68.2)
1	14 (31.8)
Current disease stage	
IIIc	1 (2.3)
IV M1a	9 (20.5)
IV M1b	11 (25.0)
IV M1c	23 (52.3)
Measurable disease and adequate baseline assessment	
Yes	37 (84.1)
No	4 (9.1)
Not reported	3 (6.8)
Number of involved disease sites	
1	4 (9.1)
2	9 (20.5)
3	8 (18.2)
4	5 (11.4)
>4	18 (40.9)
ECOG = Eastern Co-operative Oncology Group	

### Safety results

No grade 3 or greater infusion-related AEs, or other AEs preventing the full tremelimumab dose from being administered were reported. Although under-powered due to the early stopping of the clinical trial, the p-value for the primary hypothesis test was 0.099 (<0.10). Despite having enrolled less patients than was initially planned, the null hypothesis should be rejected with the risk of <0.0001 (type II error) of having reached a wrong conclusion that the infusion-related reaction rate did not exceed 5%. During study drug infusion, 5 (11%) subjects experienced 7 AEs (1 AE of diarrhea, abdominal distension, urticaria, flushing, hypertension, and 2 AEs of hypotension); all of these AEs were grade 1 or 2 and occurred during cycle 1. No SAEs occurred during infusion. There were no permanent discontinuations due to AEs occurring during study drug infusion; however, 1 subject temporarily discontinued study drug due to AEs of diarrhea and urticaria. Overall, 42 (95%) subjects experienced at least one AE, and 17 (39%) subjects at least 1 SAE. Three (7%) subjects permanently discontinued the study due to AEs. The most commonly reported AEs (Table 2) were fatigue and diarrhea. The most common grade 3 AEs were diarrhea (three subjects), and fatigue and dehydration (each two subjects). The only reported grade 4 AE was autoimmune thrombocytopenia (one subject). All of these side effects were consistent with the prior experience with anti-CTLA4 antibodies like tremelimumab and ipilimumab [2,3,5,6,10-13].

**Table 2:** Summary of adverse events (All causality)

	**All adverse events**	**Grade 3–4 adverse events**
During infusional + 1 hour	5 (14%)	0
Fatigue	22 (50%)	2 (4.5%)
Diarrhea	21 (48%)	3 (7%)
Rash	18 (41%)	0
Decreased appetite	16 (36%)	0
Nausea/Vomiting	15 (34%)	0
Pruritus	12 (27%)	0
Dehydration	2 (4.5%)	2 (4.5%)
Thrombocytopenia	1 (2%)	1 (2%)

This table includes all events that occurred in greater than 10% of patients (any grade), or that occurred in at least 2 patients at grade ≥3, or that occurred in any patient at grade 4.

### Tumor responses

**Figure 1:**
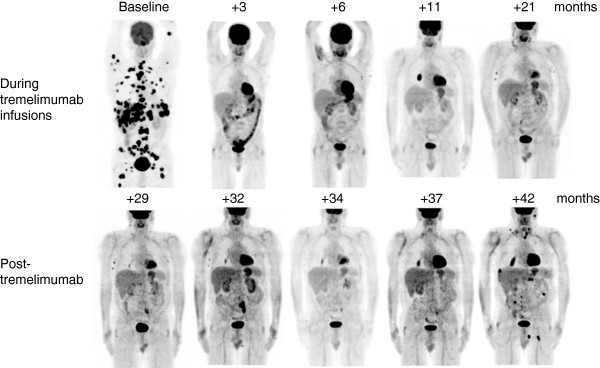
**PET scans on patient 10121002 in this trial.** The top row includes imaging scans performed while the patient was on active therapy, and the bottom row includes imaging scans off therapy. The patient had multiple FDG positive metastases at baseline that responded by the PET scan performed 3 months later and has continued on a durable response for over 3 years.

Results of antitumor activity in this study were concordant with the prior results of administering tremelimumab to patients with advanced melanoma [13]. Among 36 patients evaluable for response there were no complete responses and four partial responses (example in Figure 1), for an objective response rate of 11.1% (95% CI: [3.1%, 26.1%]) as evaluated based on the central analysis of tumor responses (Table 3). In addition, one patient had a durable partial response (over 2 years and continuing in maintained response) after an initial period of tumor progression, and another patient has remained disease-free for 2+ years after surgical resection of nodal metastases that were stable in size after starting on tremelimumab.

**Table 3:** Summary of best overall response among response evaluable patients

	**Number (%) of subjects (N=36)**
Complete response (CR)	0
Partial response (PR)	4 (11.1)
Stable for 10 weeks/no response	7 (19.4)
Objective progression	19 (52.8)
Indeterminate	6 (16.7)

### Pharmacokinetic analysis

**Figure 2:**
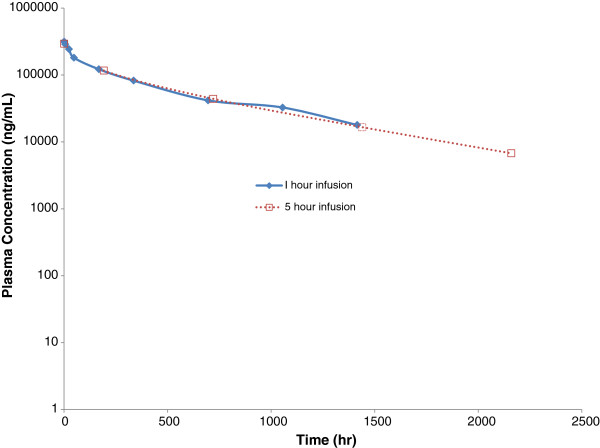
**Median tremelimumab concentration-time profiles following a 1-hour intravenous infusion compared to the 5 hour infusion.** The graph is in semi-logarithmic scale, with the 1-hour infusion data in 42 patients from study A3671022 in solid line and filled diamonds, compared to the data from the A3671008 study where 191 patients received tremelimumab with infusion times of 5–7 hours presented with a discontinuous line and open squares.

Figure 2 provides the plasma concentration over time of tremelimumab in the 42 patients who received the 1-hour infusion in study A3671022 compared to historical data from 191 patients who received the 5-hour infusion of tremelimumab in study A3671008. Tremelimumab PK profiles of the 1-hour infusion and the 5-hour infusion were similar (Table 4)_._ The mean (standard deviation [SD]) AUC_inf_ after the first dose of 1-hour intravenous infusion of 15 mg/kg tremelimumab was 104,500 (SD 32,400) μg*hr/mL. The mean (SD) C_max_ was 322 (SD 62) μg/mL and mean t_1/2_ was 470 hours (19.6 days). No subject had measurable HAHA suggesting minimal immunogenicity of tremelimumab after multiple 1-hour intravenous infusions. Taken together, these data indicate that the shorter infusion time did not alter the PK of tremelimumab, since the PK parameters are similar to those after a 5-hour infusion of tremelimumab [5,14].

**Table 4:** Summary of tremelimumab pharmacokinetic parameter values following a 1-hour intravenous infusion in comparison with those following a 5 -hour intravenous infusion in A3671008 Study

	**A3671022 Study**			**A3671008 Study**		
	**C**_**max**_	**AUC**_**inf**_(μg*h/mL)	**t**_**1/2**_	**C**_**max**_	**AUC**_**inf**_(μg*h/mL)	**t**_**1/2**_
	**(μg/mL)**		**(days)**	**(μg/mL)**		**(days)**
N	42	32	32	150	150	150
Mean	322	104500	19.6	329	112719	19.2
SD	62	32400	5.7	112	40933	5.9

## Conclusions

This study was conducted to establish the safety and tolerability of administering tremelimumab as a one-hour infusion. The use of a shorter infusion is of importance to patient convenience. The main finding of this study is that no grade 3 or greater infusion-related AEs, or other AEs preventing the full tremelimumab dose from being administered, were reported during the infusion period in any of the study subjects. The one-hour infusion safety and tolerability profile of tremelimumab was consistent with that observed to date in other studies with longer infusion times of this antibody. Therefore, we conclude that the one-hour infusion of tremelimumab is suitable for further clinical testing.

## Competing interests

Bo Huang, Erjian Wang, Poe-Hirr Hsyu, Jesus Gomez-Navarro, Diana Gerhardt and Margaret A. Marshall were employees of Pfizer at the time of this study.

## Authors’ contributions

AR, JAC, MSG, APA, TFL, DHL, BC, JAG, KL and RG treated patients and provided data. BH was the study statistician. EW and PHH performed pharmacokinetic studies, JG-N, DG and MAM were study clinicians from the sponsor. AR and MAM wrote the manuscript. All authors read and approved the final manuscript.
